# Gaze patterns during presentation of fixed and random phase radial frequency patterns

**DOI:** 10.1167/jov.21.7.2

**Published:** 2021-07-09

**Authors:** Robert J. Green, Amal Shahzad, Mazyar Fallah

**Affiliations:** 1 Centre for Vision Research, York University, Ontario, Canada; 2 Vision: Science to Applications, York University, Ontario, Canada; 3 School of Kinesiology and Health Science, York University, Ontario, Canada

**Keywords:** RF patterns, eye movements, shape and contour

## Abstract

Radial frequency (RF) patterns, circles which have had their radius modulated as a function of their polar angle, have been used in the examination of the integration of contour information around closed contour patterns. Typically, these patterns have been presented in a random orientation from trial-to-trial in order to maintain spatial uncertainty as to the location of the deformation on the pattern, as it may affect observer strategy and performance. However, the effect of fixed and random orientation (phase) on observer gaze strategies used to discriminate RF patterns has not been directly tested. This study compared fixation patterns across four conditions: fixed phase single cycle; random phase single cycle; fixed phase three cycle; and random phase three cycle RF3 patterns. The results showed that observers fixated on the known location of deformation for the fixed phase single cycle condition but used a more central fixation for the other three conditions. This strategy had a significant effect on observer thresholds for the fixed phase single cycle condition, with greater adherence to the strategy resulting in lower thresholds. It was also found that for the single cycle patterns observers tended to fixate on different locations on the pattern: on the maximum orientation difference from circular for the fixed phase pattern; and on the point of maximum curvature for the random phase pattern. These differences in gaze patterns are likely driven by the underlying local or global processing of the fixed or random phase single cycle patterns, respectively.

## Introduction

The perception of part or whole simple shapes by the visual system is thought to be a mid-level process (Merigan, 1996; Pasupathy & Connor, 2002; Yau, Pasupathy, Brincat, & Connor, 2013) in the feed forward hierarchy of human vision (Van Essen, Anderson, & Felleman, 1992). One pattern which has been useful in the study of simple shape perception has been the radial frequency (RF) pattern. An RF pattern (Wilkinson, Wilson, & Habak, 1998) is a circle with a radius which has been modified by one or more sine waves as a function of polar angle. Changing the radial frequency (RF number) changes the number of complete sine waves which can fit around the pattern. Increasing the amplitude of the sine wave or the number of sine waves present on the pattern (i.e. increasing RF number or number of cycles) increases the discriminability of the pattern. Thus, previous research modified the amplitude of the sine wave/s to find observer thresholds for a fixed number of sine waves (or cycles of modulation) around a pattern. Comparing the results for patterns with differing cycles of modulation researchers drew various conclusions about the perception of RF patterns and, more generally, mid-level visual processes. Although RF patterns do not describe all shapes necessary for object recognition (see Schmidtmann & Fruend, 2019), RF patterns are stimuli which can be used to increase our understanding of how contour information is integrated within the visual pathway. Similar to how a Gabor pattern is useful for studying the early visual pathway, RF patterns are useful for examining factors which effect “mid-level” human vision.

One of the primary conclusions about RF patterns in previous studies was that contour information was integrated around the pattern when the RF number was less than 10 (Bell, Dickinson, & Badcock, 2008; Dickinson, Almeida, Bell, & Badcock, 2010; Dickinson, McGinty, Webster, & Badcock, 2012; Hess, Wang, & Dakin, 1999; Loffler, Wilson, & Wilkinson, 2003; Schmidtmann, Kennedy, Orbach, & Loffler, 2012; Tan, Dickinson, & Badcock, 2013). One method these studies used to determine whether there was evidence for integration of contour information was the comparison of observer thresholds to those predicted by probability summation. Observer thresholds significantly lower than probability summation estimates were concluded to demonstrate evidence for integration of information.

However, Baldwin, Schmidtmann, Kingdom, and Hess (2016) noted that previous studies had used High Threshold Theory (HTT; Quick, 1974), not Signal Detection Theory (SDT; Green & Swets, 1966) to generate probability summation estimates. They found that their data was not significantly different to probability summation estimates generated by SDT and concluded that previous studies may have drawn incorrect conclusions about the integration of contour information around RF patterns.

Following this, a series of experiments (Green, Dickinson, & Badcock, 2017; Green, Dickinson, & Badcock, 2018a; Green, Dickinson, & Badcock, 2018b) investigated the methods used in Baldwin et al. (2016) to determine if there were any confounding factors and whether evidence for the integration of contour information around RF patterns existed. The main finding was that the presentation of RF patterns with a fixed phase (fixed orientation of the pattern) reduced observer thresholds, compared to random phase patterns, when the number of cycles of modulation was low (Green et al., 2017). As the cycles of modulation were increased on the pattern, there was less of an effect of the fixed phase on observer thresholds. This resulted in a flattening of the slope of integration and a lack of difference between observer performance and probability summation.

One hypothesis for explaining why a difference in performance was found for fixed phase and random phase RF patterns was that observers knew where the deformation would occur on the pattern. They could then follow different viewing strategies for the two patterns: for the random phase patterns, as they were unsure of where the deformation would occur, fixate close to the center of the pattern; for the fixed phase patterns, as they knew deformation would occur at a specific location, fixate at that location. However, this hypothesis was not directly tested with eye tracking and is therefore the aim of the current study.

The current study will use RF3 patterns (as used in Green et al., 2017) with one and three cycles of modulation. It was hypothesized that our results would replicate those of Green et al. (2017) and find a significant difference between fixed and random phase patterns at one cycle of modulation, but not three cycles of modulation. It was also hypothesized that naïve participants without any information about the difference between the two conditions would quickly develop different viewing strategies for fixed and random phase patterns at one cycle of modulation. Specifically, observers would fixate at the location of deformation when viewing a fixed phase RF3(1) and they would show a more uniform (or central) distribution of fixations when viewing a random phase RF3(1), and fixed and random phase RF3(3).

## Methods

### Observers

Twenty undergraduate students at York University, Toronto, participated in the current study. All were naïve to the aims of the experiment and had normal or corrected-to-normal vision, which was assessed using a Snellen chart. Informed consent was obtained prior to the experiment. Research was approved by York University's Human Participants Review Committee and conforms to the Declaration of Helsinki.

### Stimuli

The stimuli were RF patterns (Wilkinson et al., 1998), circular contours which have their radius (*R*) defined as a function of polar angle (θ):

(1) $$\begin{array}{c} R\left(\theta \right)=R_{0}\times \left(1+A\sin\left(\omega \theta +\varphi \right)\right)\; \end{array}$$

where *R*_0_ is the radius of the unmodulated circle (2 degrees of visual angle), *A* is the amplitude of the sine wave (as a proportion of *R*_0_), ω is the RF number (3), which defines the radial frequency by setting the number of complete sine waves that can fit within 2π radians, and ϕ is the phase (orientation) of the pattern. Two patterns were used: an RF3 with all three cycles of modulation present (RF3); and an RF3 with one cycle of modulation present (RF3(1)). As established by Loffler et al. (2003), for patterns with one cycle of modulation (i.e. RF3(1)), the modulated portion of the contour conforms solely to a first derivative of a Gaussian (D1) with a slope and amplitude identical to that of the sine waves used for completely modulated patterns (i.e. RF3). Using a sine wave may result in unintended local cues which the observer may use to differentiate the RF pattern and circle. In other words, at one cycle of modulation a D1, not a sine wave, is used to modulate the pattern's radius, which provides a smooth transition the modulated and unmodulated portions of the pattern. The cross-sectional luminance profile of the contour was defined by the fourth derivative of a Gaussian (D4) with a peak spatial frequency of 8 c/deg. The center of the pattern was jittered randomly within a 20′ box in the center of the screen.

### Apparatus

Stimuli were created using MATLAB (Mathworks, Nantucket, MA, USA, 2010) and presented using Presentation software (Neurobehavioral Systems, Inc., Berkeley, CA, USA) on a 21-inch cathode ray tube monitor (1024 × 768 pixels; 60 Hz). Gaze location was recorded using EyeLink II (infrared, 500 Hz; SR Research Ltd., Ontario, Canada) and was calibrated at the start of each block and drift corrected as required during testing. A viewing distance of 57 cm was maintained using a chin rest.

### Procedure

**Figure 1:**
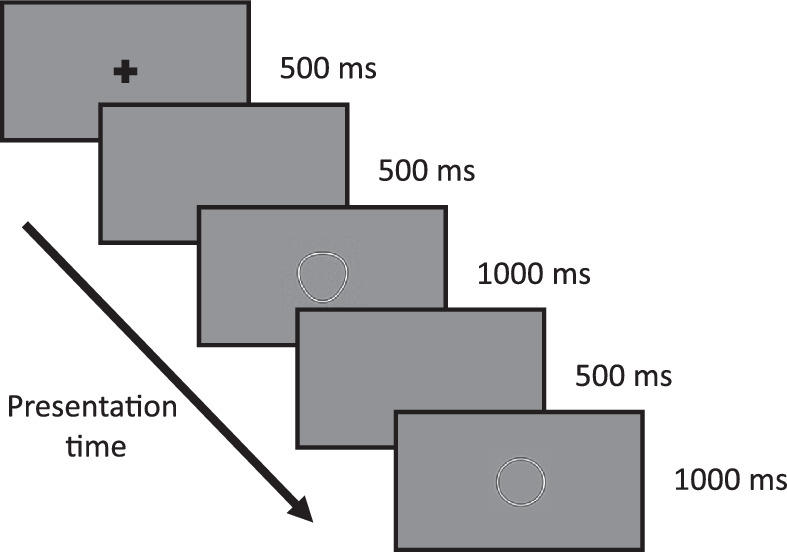
. Task paradigm. Note that fixation cross is only present at the beginning of the trial.

A two-alternative-forced-choice (2AFC) task was used for all conditions and eye movements were recorded throughout the entire testing block. One interval contained a circle (*A* = 0 in Equation 1) and the other an RF pattern, with the order of presentation randomized between trials. Each trial consisted sequentially of a 500 ms fixation cross, 500 ms blank screen, 1000 ms presentation of interval one, 500 ms blank screen, 1000 ms presentation of interval two, and ending with response collection (see Figure 1). The observer indicated which interval contained the pattern most deformed from circular by clicking either the left (first interval) or right (second interval) mouse button. At the beginning of each testing block observers were instructed to “always look at the fixation cross when it is on-screen; when it is gone you may look anywhere you like.”

**Figure 2.**
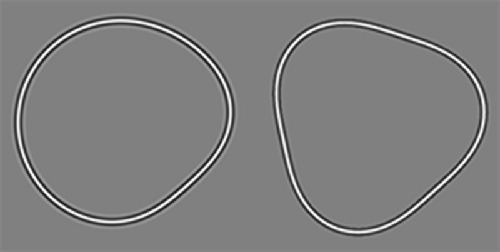
Left, RF3(1) and right, RF3. Both patterns are presented in the orientation shown for the fixed phase condition.

There were four conditions: RF3(1) fixed; RF3(1) random; RF3 fixed; and RF3 random. Each condition was tested twice using a three-down one-up staircase procedure and the order of conditions was randomized between participants. For the fixed phase condition, the patterns were always presented in the same orientation (φ=0; see Figure 2). For the random phase, a random integer (*n*) from 1 to 20 was used in the equation $\varphi =\frac{2\pi n}{20}$ for RF3(1) and $\varphi =\frac{2\pi n}{60}$ for RF3 so that observers would not be able to anticipate the location of deformation on the circle. The equations differ because unique orientations of an RF3 are only possible from 0 to $\frac{2\pi }{3}$ radians.

### Analysis

Eye movement data was first removed of all blinks and the 150 ms before and after the blink. The center of the screen was adjusted each trial by subtracting the location of the observer's fixation during the presentation of the fixation cross. Fixations were identified as a 50 ms window which does not exceed 20 degrees/s and is immediately preceded by an eye movement greater than 20 degrees/s. To analyze all the eye movements made within the 1000 ms presentation of the stimulus, all the fixations were converted into vectors relative to the center of the stimulus. The phase of the stimulus for each trial was subtracted from the polar angle of the observer's fixations, thereby normalizing the eye movements across all conditions. These vectors were summed using the formulae outlined in Berens (2009) to produce the resultant vector for that trial. Circular statistics (Berens, 2009) was then performed to determine the polar angle and peakedness (circular kurtosis; *k*) of each observer's testing block. Higher values of *k* indicate observers are fixating more in one area than any other.

For all analyses, Cook's distance scores greater than three times the mean were identified as outliers and removed from the linear mixed effect models (LMMs). This was a more conservative method for our data than identifying scores greater than $\frac{4}{n}$ as outliers and resulted in the removal of between one and four data points each analysis, with two being the typical number. For all LMMs participants and repetition of condition (first and second) were random factors in the model. To make the LMM output more readable, a Satterthwaite approximation was used (Luke, 2017). For analysis of peakedness in the single cycle condition, it was predicted that a negative relationship would exist between peakedness and thresholds for the fixed phase condition and there would be a positive relationship between peakedness and thresholds for the random phase condition. Thus, for these analyses a one-tailed distribution was used.

## Results

**Figure 3.**
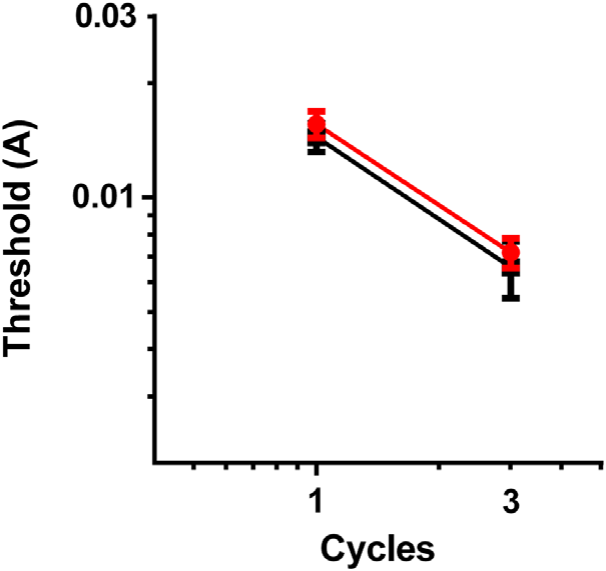
Geometric means with 95% confidence intervals of observer thresholds for fixed (red) and random (black) phase conditions at one and three cycles of modulation.

It is apparent from Figure 3 that there was no difference in observer thresholds for fixed and random phase conditions at one and three cycles of modulation. An extra sum of squares f-test found that one curve adequately described both data sets, *F*(2,156) = 1.48, *p* = 0.23.

### RF3(1)

**Figure 4.**
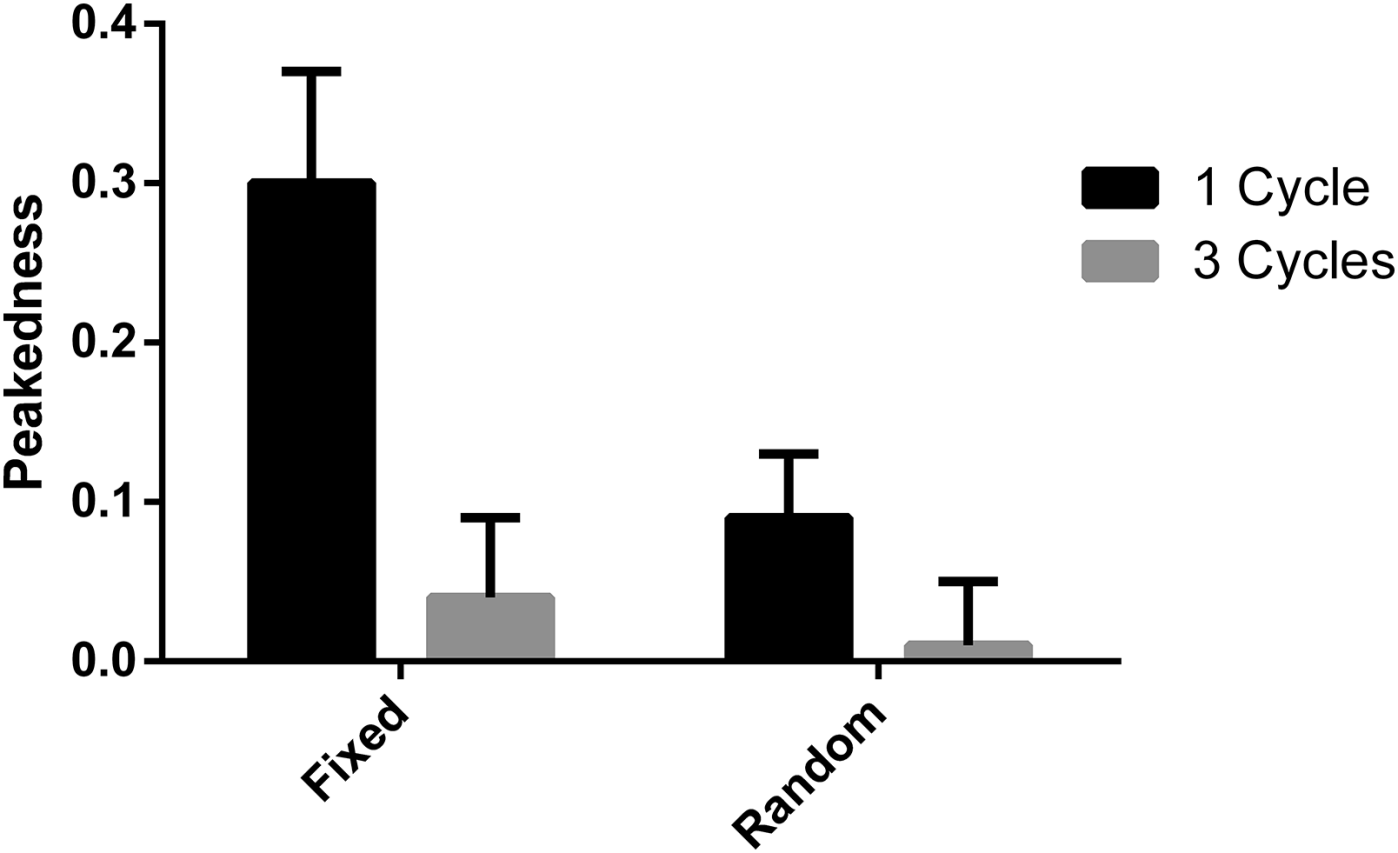
Peakedness of observer fixations for all four conditions with 95% confidence intervals.

At one cycle of modulation, fixations in the fixed phase condition were significantly more peaked (nonuniform in their distribution; M = 0.30, 95% confidence interval; [CI] = 0.23 to 0.37) than fixations in the random phase condition (M = 0.09, 95% CI = 0.05 to 0.13), *t*(54.81) = 7.42, *p* < 0.001, BF = 3.34*10^4^ (see Figure 4). Therefore, we investigated the effect of an observer's peakedness and polar angle of fixation on their threshold for detection. There was a significant negative effect of peakedness *t*(31.44) = −1.80, *p* = 0.04, BF = 1.71 (one tailed) and a significant positive effect of polar angle *t*(30.29) = 2.59, *p* = 0.01, BF = 3.15 on thresholds for the fixed phase condition. For the random phase condition, there was a significant positive effect of peakedness on thresholds, *t*(21.19) = 2.98, *p* < 0.001, BF = 12.68 (one tailed) but no significant effect of polar angle *t*(26.53) = −0.60, *p* = 0.55, BF = 0.27 on thresholds.

**Figure 5.**
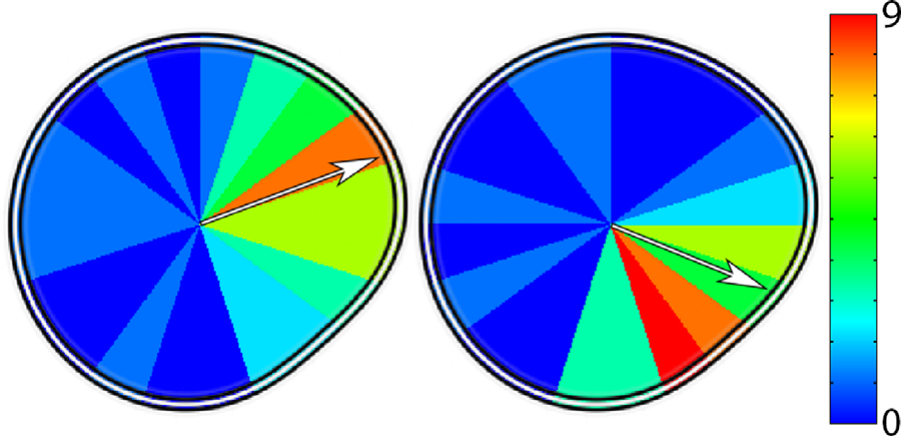
Heatmaps of fixation locations for random phase (left) and fixed phase (right) single cycle RF3s. The fixation locations are binned in π/10 radian segments and contain the total number of observers with their overall fixation vector for that condition within that bin. The white arrow shows the vector sum of all the observer fixations during stimulus presentation. The data shows that observers display different fixation locations depending on whether the pattern is fixed or random phase.

The standard deviation of the polar angle of the fixation vectors were calculated for the first 15 trials – the average number of trials before an incorrect response and considered to be suprathreshold patterns. There was a significantly higher amount of variation in fixation vectors for the random phase condition than the fixed phase condition *t*(52.65) = 7.12, *p* < 0.001, BF = 1.96*10^4^. Figure 5 shows the mean polar angle of all observers’ mean fixation position for both conditions. Note that for the random phase condition (left) observers seem to favor the peak of the lobe (i.e. the point of maximum radius), whereas for the fixed phase condition (right) observers favor the maximum deviation from circular (i.e. the zero crossing of the sine wave).

### RF3

To analyze results for three cycles of modulation, the polar angle was multiplied by three within a negative-pi to positive-pi circle. This resulted in all three cycles of modulation overlaying the same position in polar space. After the vector calculations the polar angle is divided by three to make the pattern equivalent to a single cycle pattern. Therefore, if an observer made three eye movements, one to the peak of each lobe of the pattern (i.e. the approximate position depicted in the left portion of Figure 5) their resultant vector would be a length of 1 with a polar angle equal to the peak of the sine wave ($-\frac{\pi }{6}$ for our data), rather than a vector length of 0.

There was no significant difference in the peakedness of observer eye-movements when comparing the fixed phase (M = 0.04, 95% CI = −0.003 to 0.09) and random phase conditions (M = 0.01, 95% CI = −0.03 to 0.05), *t*(70.00) = 1.46, *p* = 0.15, BF = 0.58. Observer thresholds were not affected by peakedness, *t*(32.90) = 0.34, *p* = 0.73, BF = 0.24, or polar angle *t*(32.22) = 0.33, *p* = 0.75, BF = 0.24, for the fixed phase condition. Similarly, for the random phase condition, peakedness *t*(24.73) = 0.71, *p* = 0.49, BF = 0.29, and polar angle *t*(20.70) = 0.68, *p* = 0.51, BF = 0.28, had no effect on thresholds. Following the analysis of the single cycle patterns, the standard deviation of the polar angle of the fixation vectors were calculated for the first 15 trials. There was a significantly higher amount of variation in fixation vectors for the random phase condition than the fixed phase condition *t*(68.00) = 5.92, *p* < 0.001, BF = 2.08*10^3^.

### Reference pattern

**Figure 6.**
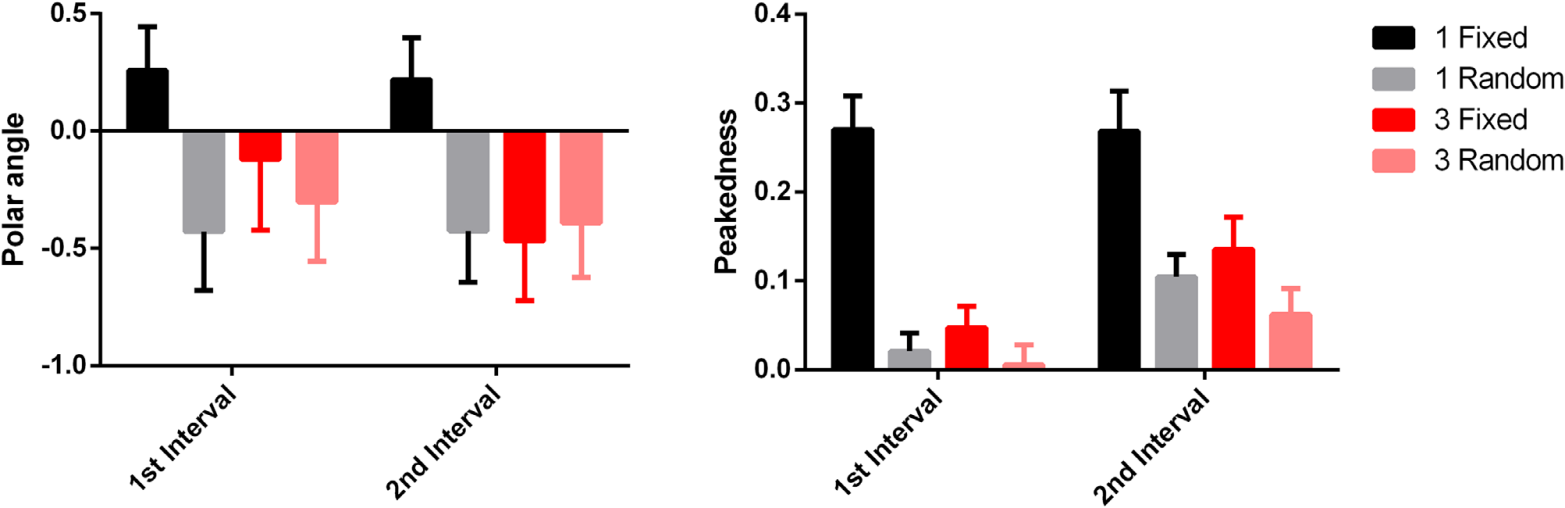
Mean polar angles and peakedness for fixational eye movements occurring during the presentation of the reference stimulus in either the first or second interval. The fixed phase condition at 1 cycle of modulation clearly displays different fixational eye movements in comparison to the other three conditions.

To better understand gaze strategies that participants employed, the eye movements during the presentation of the reference stimulus were also analyzed. Figure 6 shows the group means with standard error bars for fixational eye movements during the presentation of the reference stimulus (circle). The metrics are shown for when the reference stimulus is presented in the first or second interval. It is clear from the graph that the fixed phase single cycle of modulation condition is different from the other conditions, and that there were some differences between the first and second interval in the other three conditions Three linear mixed models were used to analyze the effect of condition and interval on both polar angle and peakedness. For polar angle there was no significant effect of condition, *t*(297.00) = −1.32, *p* = 0.19, BF = 0.49, or interval, *t*(297.00) = 0.39, *p* = 0.70, BF = 0.24. For peakedness, there was a significant effect of both condition, *t*(297.00) = −5.40, *p* < 0.001, BF = 7.54*10^2^, and interval, *t*(297.00) = 5.55, *p* < 0.001, BF = 1.01*10^3^. Bonferroni adjusted pairwise comparisons showed the fixed phase, one cycle of modulation condition had a significantly more peaked distribution of fixational eye-movements than all other three conditions (*p* < 0.05). There were no other differences in peakedness between conditions (*p* > 0.05). For fixed phase at one cycle of modulation and random phase at three cycles of modulation, there was no significant difference in peakedness when the reference stimulus appeared in either the first or second interval (*p* > 0.05). For random phase at one cycle of modulation and fixed phase at three cycles of modulation the reference stimulus appearing in the second interval had a significantly higher peakedness compared to when it was shown in the first interval (*p* < 0.05).

**Figure 7.**
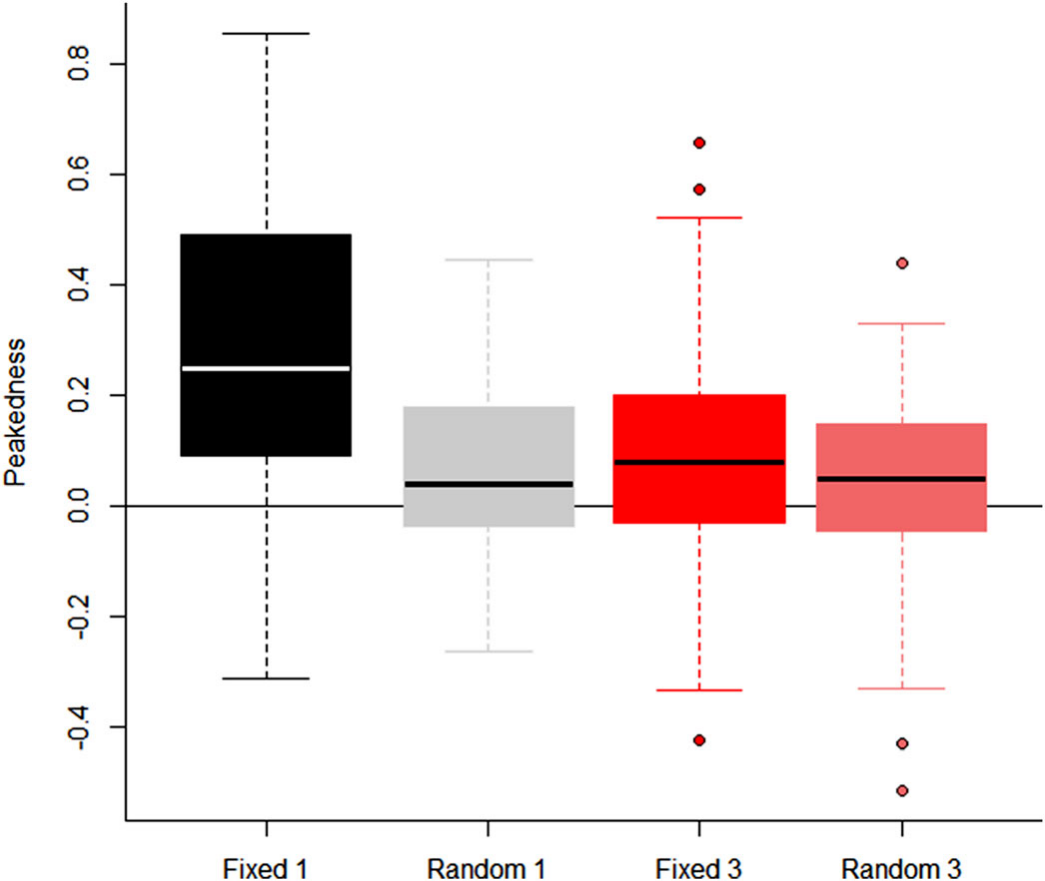
Box and whisker plots of the overall peakedness of fixational eye movements during the presentation of the reference stimulus.

Figure 7 shows the range of peakedness during the presentation of the reference stimulus for both intervals combined. The range of peakedness in observer fixations for the fixed phase single cycle RF pattern condition is greater than the other conditions.

## Discussion

The aim of the current study was to investigate the spontaneous strategies that observers develop when viewing fixed phase and random phase RF patterns. For the single cycle pattern (RF3(1)), our results for the target stimulus suggested that observers adopted different strategies for observing fixed and random phase patterns. Observer thresholds decreased (i.e. performance improved) when fixations were more peaked for the fixed phase condition and observer thresholds increased (i.e. performance decreased) when fixations were more peaked for the random phase condition. In other words, for fixed phase patterns the best observer strategy was to look at the location of deformation, whereas for the random phase patterns the best strategy was to look at the center of the pattern. If there is local processing, we would expect an improvement in performance when looking at the location containing information, in this case the location of deformation. Given we find an improvement in performance when fixating on deformation for the fixed phase single cycle condition, but the opposite for the random phase single cycle condition, we conclude there is evidence of local processing and integration for fixed and random phase single cycle RF3s, respectively.

Given that these results were found within their respective conditions, there is no question about spatial certainty vs uncertainty. The grouped results were the same for fixed (spatially certain) and random phase (spatially uncertain) patterns at one cycle of modulation (see Figure 3). However, when investigating the effects of observer gaze within these conditions, it was found that observers within the fixed phase condition achieved better results when looking at the location of deformation compared to when looking centrally. Therefore, optimal performance was not based on simply knowing where the information was (spatially certainty), observers also needed to look at the location of deformation to achieve lower thresholds.

For the patterns with three cycles of modulation (RF3(3)), there was no effect of polar angle or peakedness on observer thresholds for fixed phase or random phase patterns. This suggests there is no difference in the viewing strategies employed by the observers for the random phase and fixed phase conditions. Therefore, when there are three cycles of modulation, regardless of whether the observer knows where the deformation is going to occur, observers adopt a central fixation strategy.

To better understand the observer strategies, results from the eye movement data collected during the presentation of the reference stimulus was analyzed. Of particular interest is the behavior observers are displaying during the first interval. Because of the randomization of the order of stimulus presentation, observers do not know whether the target stimulus will be in the first or second interval. Thus, the behavior displayed during the first interval likely describes their strategy for target detection. This clearly shows a more peaked eye movement distribution for the fixed phase, single cycle RF pattern. There was no difference between the other conditions and, therefore, it suggests that when the observers are unsure of which stimulus (reference or test) will be presented, they fixate on the location of deformation for the fixed phase single cycle RF pattern and on the center for all other conditions.

The combination of the eye movement data for the test and reference stimulus provides strong evidence that observers, without any previous experience with RF patterns, develop different viewing strategies when observing fixed phase and random phase single cycle patterns. They also support the hypothesis that observers would fixate on the location of deformation for fixed phase patterns and more centrally for random phase patterns and that these strategies would produce the best results. However, this begs the question as to why there was no significant difference found between the fixed phase and random phase conditions at one cycle of modulation. We suggest the answer to this question is simply the consistency of the use of strategies across observers. The current study used observers who had not previously encountered RF patterns and were not given any instruction on the pattern's orientation. This was done to measure whether observers would spontaneously develop different viewing strategies for the different conditions but may have led to inconsistency in how these strategies were applied by different participants. The wide range of peakedness found in the fixed phase single cycle condition (see Figure 7) indicates a wider variety of adherence to the strategy of fixating on the deformation on the pattern. Some observers had a highly peaked distribution of fixations, suggesting they spent most of their time fixating where the deformation appeared. Whereas others had a low peakedness, approximating that of the other conditions and indicating a more central fixation during presentation. Given the significant effect of peakedness on thresholds, it is likely that this variation between observers resulted in an overall lack of difference in thresholds between the two conditions.

Previous research investigated the involvement of V4 neurons in eye gaze using single cell recording in monkeys (Moore, 1999). Their results found presaccadic re-activation of orientation selective V4 neurons, which is indicative of their involvement in the guidance of the saccade. As V4 also has curvature selectivity (Pasupathy & Connor, 1999), V4 neurons could similarly guide saccade endpoints to portions of the radial frequency pattern. Therefore, we would expect that the segment of the pattern that is used by participants to perform the discrimination task would be the most active curvature representation in the V4 retinotopic map, and thus guide saccades to that location.

There is evidence for curvature being important in the perception of shapes (see Schmidtmann, Jennings, & Kingdom, 2015), however, for RF patterns with continuous contours, there is evidence suggesting that maximum deviation from circular is the key to discriminating these patterns from circles. Dickinson et al. (2012) found that RF patterns with differing frequency but with the same number cycles of modulation had the same maximum deviation from circularity at their thresholds for detection. This evidence is strengthened by the research using rectified RF patterns (where the sine wave has been rectified such that the amplitude remains completely positive or completely negative) and conventional RF patterns (Dickinson, Cribb, Riddell, & Badcock, 2015). These rectified patterns create points of infinite “positive” (convex) curvature and points of infinite “negative” (concave) curvature for the positive and negative rectified patterns, respectively. Results showed that at their threshold for detection both the rectified and conventional RF patterns had the same maximum deviation from circular. If curvature was the key salient feature, the points of infinite curvature should have resulted in significantly different thresholds for the rectified patterns compared to the conventional ones. This result has been further corroborated by Dickinson, Haley, Bowden, and Badcock (2018) who found that performance in visual search tasks is poor when trying to identify RF patterns amongst rectified RF patterns of the same frequency, implying that absolute measures of curvature are less important to shape analysis than the relative positions of corners on the patterns. Such corners might be inferred through the extrapolation of tangents to the points of maximum deviation from circular. If maximum deviation from circular is of importance in discrimination of RF patterns, we would expect that when observers are using a local processing strategy, saccade endpoints would cluster around this location on the RF pattern. However, when participants cannot use a local processing strategy, saccade end points would be guided elsewhere.

The heatmap of fixation vectors and the overall fixation vector displayed in Figure 5 clearly shows a difference in observer fixations between the fixed phase and random phase conditions at one cycle of modulation. For the fixed phase patterns (see Figure 5 right) the fixations are clustered around the maximum deviation from circular on the pattern. This would suggest a local processing strategy, as a high saliency point is guiding eye movements. It also suggests, that because thresholds decrease (i.e. performance increased) when peakedness was higher, that this strategy was effective. This gives further support to the suggestion that maximum orientation deviation from circular, are the points which contain the greatest signal (Dickinson et al., 2015; Dickinson et al., 2012). For the random phase patterns (see Figure 5 left) the fixations are clustered more around the peak of the lobe (referred to by Loffler et al., 2003 as the point of maximum curvature) and not the maximum orientation deviation from circular. This would suggest a global processing strategy, as a shape cue – curvature is guiding observer gaze. Contrary to the fixed phase results, thresholds increased when observers had a more peaked distribution of fixations. This means that performance decreased when observers looked at the point of maximum curvature and lends further support to the maximum orientation deviation from circular having the greatest local signal cue on the RF pattern. It should be noted again, that the peakedness of the distribution of eye movements in the random phase single cycle condition was relatively low compared to the fixed phase condition, meaning observers tended to fixate more in the center of the pattern.

The comparison of eye movements made during the first interval and second interval for the presentation of the reference stimulus gives us insight into what strategy observers are using after they have seen the test stimulus. Eye movements during the first interval indicate the strategy used when the observer does not know what stimulus will be presented, however, considering only correct trials were analyzed, it is likely that for reference stimuli presented in the second interval observers had already identified that the test pattern was in the first interval. For the fixed phase single cycle and the random phase three cycle patterns, there was no change in peakedness, but for the random phase single cycle and the fixed phase three cycle patterns there was an increase in peakedness. This might indicate that observers, after identifying the location of deformation on the RF pattern in the first interval, seek to gather further information from that location on the reference pattern. Again, this fixation tended to be at the peak of the lobe and not the maximum deviation from circular.

The current study examined how fixational eye movements were affected when viewing fixed and random phase RF patterns with one and three cycles of modulation. There was strong evidence that eye movements were significantly different for the fixed phase single cycle RF patterns, with observers tending to fixate on the deformation on the pattern. For the other three conditions, fixations were relatively central. These results suggest that knowing the location of deformation for single cycle RF patterns changed observer strategies of naïve (having not previously encountering RF patterns) participants and resulted in lower detection thresholds (i.e. better performance) which supports the hypothesis put forth by Green et al. (2017). It was also found that observers look at different parts of the pattern when viewing a single cycle of modulation in either random or fixed phase. These gaze patterns reflected the strategies used by the observers which was driven by either global or local processing of the random or fixed phase pattern, respectively.
